# *N*-(2-Chloro-2-nitro-1-phenyl­prop­yl)-4-methyl­benzene­sulfonamide

**DOI:** 10.1107/S1600536807067712

**Published:** 2008-01-04

**Authors:** Sanjun Zhi, Tengfei Li, Guanghui An, Yi Pan

**Affiliations:** a School of Chemistry and Chemical Engineering, State Key Laboratory of Coordination Chemistry, Nanjing University, Nanjing 210093, People’s Republic of China

## Abstract

In the title compound, C_16_H_17_ClN_2_O_4_S, the dihedral angle between the phenyl and benzene rings is 19.4 (2)°. The crystal packing is stabilized by inter­molecular N—H⋯O hydrogen bonds, as well as by intra- and inter­molecular C—H⋯O hydrogen bonds.

## Related literature

For general background, see Kemp (1991 ▶); Qui & Silverman (2000 ▶); Orlek & Stemp (1991 ▶), Han *et al.* (2007 ▶); Li *et al.* (2007 ▶). For bond-length data, see: Allen *et al.* (1987 ▶).
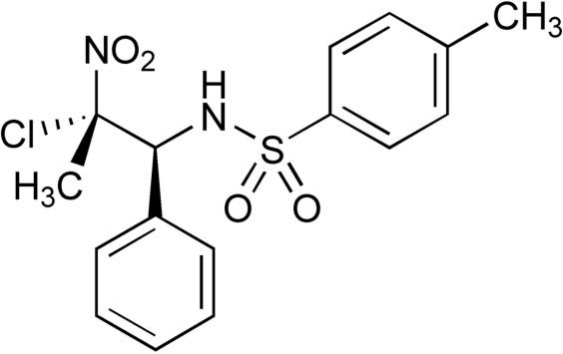


## Experimental

### 

#### Crystal data

C_16_H_17_ClN_2_O_4_S
*M* *_r_* = 368.83
Orthorhombic, 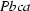

*a* = 7.8254 (8) Å
*b* = 19.610 (2) Å
*c* = 22.533 (3) Å
*V* = 3457.8 (7) Å^3^
*Z* = 8
Mo *K*α radiation
μ = 0.36 mm^−1^
*T* = 291 (2) K
0.30 × 0.26 × 0.24 mm

#### Data collection

Bruker SMART APEX CCD area-detector diffractometer
Absorption correction: multi-scan (*SADABS*; Bruker, 2000 ▶) *T*_min_ = 0.901, *T*_max_ = 0.921
17527 measured reflections
3396 independent reflections
2467 reflections with *I* > 2σ(*I*)
*R*_int_ = 0.070

#### Refinement

*R*[*F* ^2^ > 2σ(*F* ^2^)] = 0.056
*wR*(*F* ^2^) = 0.125
*S* = 1.01
3396 reflections
222 parameters
H atoms treated by a mixture of independent and constrained refinement
Δρ_max_ = 0.28 e Å^−3^
Δρ_min_ = −0.26 e Å^−3^

### 

Data collection: *SMART* (Bruker, 2000 ▶); cell refinement: *SAINT* (Bruker, 2000 ▶); data reduction: *SAINT*; program(s) used to solve structure: *SHELXTL* (Bruker, 2000 ▶); program(s) used to refine structure: *SHELXTL*; molecular graphics: *SHELXTL*; software used to prepare material for publication: *SHELXTL* and *PLATON* (Spek, 2003 ▶).

## Supplementary Material

Crystal structure: contains datablocks I, global. DOI: 10.1107/S1600536807067712/at2518sup1.cif

Structure factors: contains datablocks I. DOI: 10.1107/S1600536807067712/at2518Isup2.hkl

Additional supplementary materials:  crystallographic information; 3D view; checkCIF report

## Figures and Tables

**Table 1:** Hydrogen-bond geometry (Å, °)

*D*—H⋯*A*	*D*—H	H⋯*A*	*D*⋯*A*	*D*—H⋯*A*
C3—H3⋯O1	0.98	2.46	2.933 (4)	109
C3—H3⋯O3	0.98	2.42	2.779 (4)	101
C1—H1*A*⋯O2^i^	0.96	2.52	3.369 (4)	147
C1—H1*B*⋯O1^ii^	0.96	2.58	3.310 (4)	133
N2—H2*A*⋯O2^i^	0.89 (2)	2.32 (3)	3.141 (3)	153

Symmetry codes: (i) 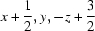; (ii) 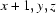.

**Fig. 1.**
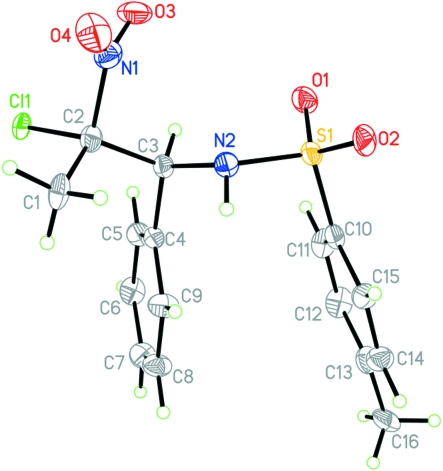
The molecular structure of (I), with atom labels and 30% probability displacement ellipsoids for non-H atoms.

## supplementary crystallographic information

### Comment

Since the vicinal haloamines are important building blocks in organic and medicinal chemistry, many attentions are attracted to the aminohalogenation reactions of functionalized alkenes (Kemp, 1991; Qui & Silverman, 2000). In recent years, many new aminohalogenation processes of several kinds of functionalized alkenes have been developed (Han *et al.*, 2007), with the different nitrogen/halogen sources in the presence of metallic catalysts (Li *et al.*, 2007). However, the aminohalogenation reactions of 2-nitro-propenyl benzene was not been well documented. Recently, we synthesized the title compound (I) from 2-nitro-propenyl benzene. As part of this study, we have undertaken the X-ray crystallographic analysis of (I) in order to elucidate the conformation and configuration of this product.

The bond lengths and angles in (I) are in good agree with expected values (Allen *et al.*, 1987). The dihedral angle between the phenyl and benzene rings is 19.4 (2)°. The packing is stabilized by intermolecular N—H···O as well as intra and intermolecular C—H···O interactions in the crystal structure (Table 1).

### Experimental

*N*-cChlorosuccinimide (400 mg, 3.0 mmol) was added into a solution of MnSO_4_ (30.2 mg, 0.20 mmol), 1-benzyl-2-nitro-propene (163 mg, 1 mmol), tolunesulfonamide (513 mg, 3 mmol) and 4 Å molecular sieves (500 mg) in CH_2_Cl_2_ (5.0 ml) with nitrogen atmosphere. The resulting mixture was stirred at room temperature for 48 h. Reaction was quenched with saturated aqueous Na_2_S_2_O_3_ solution. The solid precipitates were filtered off and washed with ethyl acetate (3 × 10 ml). The organic solution was concentrated and then purified *via* flash chromatography (ethyl acetate/ hexane, 1:4, *v*/*v*) provide the title compound (I) as white solid (276 mg) in yield of 75%. A colourless crystal of (I) for X-ray analysis was obtained by slow evaporation from ethyl acetate solution system.

### Refinement

The H atom bonded to N was located in a difference map and refined with restraint of N—H = 0.89 (3) Å, and with *U*_iso_(H) = 1.2*U*_eq_(N). Other H atoms were geometrically placed and were treated as riding, with C—H distances of 0.93, 0.96 and 0.98 Å for aromatic, methyl and methine H atoms, respectively, and with *U*_iso_(H) = 1.2*U*_eq_(aromatic and methyne C) or 1.5*U*_eq_(methyl C).

### Crystal data

**Table:**

C_16_H_17_ClN_2_O_4_S	*D*_x_ = 1.417 Mg m^−^^3^
*M**_r_* = 368.83	Melting point: 423.2 K
Orthorhombic, *P**b**c**a*	Mo *K*α radiation λ = 0.71073 Å
Hall symbol: -P 2ac 2ab	Cell parameters from 7468 reflections
*a* = 7.8254 (8) Å	θ = 2.3–27.9º
*b* = 19.610 (2) Å	µ = 0.36 mm^−^^1^
*c* = 22.533 (3) Å	*T* = 291 (2) K
*V* = 3457.8 (7) Å^3^	Block, colourless
*Z* = 8	0.30 × 0.26 × 0.24 mm
*F*_000_ = 1536	

### Data collection

**Table:**

Bruker SMART APEX CCD area-detector diffractometer	3396 independent reflections
Radiation source: sealed tube	2467 reflections with *I* > 2σ(*I*)
Monochromator: graphite	*R*_int_ = 0.070
*T* = 291(2) K	θ_max_ = 26.0º
φ and ω scans	θ_min_ = 2.1º
Absorption correction: multi-scan(SADABS; Bruker, 2000)	*h* = −9→8
*T*_min_ = 0.901, *T*_max_ = 0.921	*k* = −24→24
17527 measured reflections	*l* = −16→27

### Refinement

**Table:**

Refinement on *F*^2^	Secondary atom site location: difference Fourier map
Least-squares matrix: full	Hydrogen site location: inferred from neighbouring sites
*R*[*F*^2^ > 2σ(*F*^2^)] = 0.056	H atoms treated by a mixture of independent and constrained refinement
*wR*(*F*^2^) = 0.125	*w* = 1/[σ^2^(*F*_o_^2^) + (0.06*P*)^2^ + 0.58*P*] where *P* = (*F*_o_^2^ + 2*F*_c_^2^)/3
*S* = 1.01	(Δ/σ)_max_ < 0.001
3396 reflections	Δρ_max_ = 0.28 e Å^−^^3^
222 parameters	Δρ_min_ = −0.26 e Å^−^^3^
Primary atom site location: structure-invariant direct methods	Extinction correction: none

### Special details

**Table:**

Geometry. All e.s.d.'s (except the e.s.d. in the dihedral angle between two l.s. planes) are estimated using the full covariance matrix. The cell e.s.d.'s are taken into account individually in the estimation of e.s.d.'s in distances, angles and torsion angles; correlations between e.s.d.'s in cell parameters are only used when they are defined by crystal symmetry. An approximate (isotropic) treatment of cell e.s.d.'s is used for estimating e.s.d.'s involving l.s. planes.
Refinement. Refinement of *F*^2^ against ALL reflections. The weighted *R*-factor *wR* and goodness of fit *S* are based on *F*^2^, conventional *R*-factors *R* are based on *F*, with *F* set to zero for negative *F*^2^. The threshold expression of *F*^2^ > σ(*F*^2^) is used only for calculating *R*-factors(gt) *etc*. and is not relevant to the choice of reflections for refinement. *R*-factors based on *F*^2^ are statistically about twice as large as those based on *F*, and *R*- factors based on ALL data will be even larger.

### Fractional atomic coordinates and isotropic or equivalent isotropic displacement parameters (Å^2^)

**Table:**

	*x*	*y*	*z*	*U*_iso_*/*U*_eq_	
C1	0.7916 (4)	0.10320 (17)	0.61740 (13)	0.0374 (7)	
H1A	0.7709	0.1030	0.6594	0.056*	
H1B	0.8606	0.1420	0.6072	0.056*	
H1C	0.8503	0.0621	0.6064	0.056*	
C2	0.6185 (4)	0.10723 (16)	0.58376 (13)	0.0333 (7)	
C3	0.5059 (4)	0.16923 (14)	0.59888 (13)	0.0292 (6)	
H3	0.3995	0.1645	0.5763	0.035*	
C4	0.5853 (3)	0.23727 (15)	0.58138 (13)	0.0293 (6)	
C5	0.5405 (4)	0.26704 (17)	0.52790 (13)	0.0362 (7)	
H5	0.4651	0.2445	0.5028	0.043*	
C6	0.6055 (4)	0.32954 (17)	0.51103 (14)	0.0415 (8)	
H6	0.5765	0.3484	0.4745	0.050*	
C7	0.7156 (4)	0.36407 (18)	0.54968 (15)	0.0437 (8)	
H7	0.7570	0.4070	0.5396	0.052*	
C8	0.7623 (4)	0.33466 (18)	0.60226 (15)	0.0444 (8)	
H8	0.8381	0.3572	0.6273	0.053*	
C9	0.6972 (4)	0.27113 (17)	0.61859 (14)	0.0414 (8)	
H9	0.7290	0.2516	0.6545	0.050*	
C10	0.3185 (3)	0.28495 (15)	0.69070 (12)	0.0314 (6)	
C11	0.2653 (4)	0.32776 (18)	0.64579 (14)	0.0402 (7)	
H11	0.2085	0.3099	0.6131	0.048*	
C12	0.2959 (5)	0.39679 (18)	0.64904 (15)	0.0465 (8)	
H12	0.2601	0.4252	0.6184	0.056*	
C13	0.3804 (4)	0.42454 (18)	0.69822 (14)	0.0419 (8)	
C14	0.4350 (4)	0.38054 (17)	0.74268 (15)	0.0423 (8)	
H14	0.4939	0.3980	0.7751	0.051*	
C15	0.4037 (4)	0.31171 (16)	0.73966 (13)	0.0349 (7)	
H15	0.4392	0.2831	0.7702	0.042*	
C16	0.4064 (4)	0.49975 (18)	0.70326 (17)	0.0491 (9)	
H16A	0.3241	0.5184	0.7304	0.074*	
H16B	0.3922	0.5204	0.6650	0.074*	
H16C	0.5196	0.5087	0.7177	0.074*	
Cl1	0.65350 (9)	0.10276 (4)	0.50665 (3)	0.03580 (19)	
N1	0.5083 (4)	0.04372 (13)	0.59707 (12)	0.0407 (6)	
N2	0.4620 (3)	0.16217 (13)	0.66251 (11)	0.0334 (6)	
H2A	0.547 (4)	0.1794 (17)	0.6841 (15)	0.040*	
O1	0.1557 (3)	0.18367 (12)	0.64347 (10)	0.0439 (6)	
O2	0.2635 (3)	0.17162 (12)	0.74582 (9)	0.0432 (6)	
O3	0.3667 (3)	0.04155 (12)	0.57591 (12)	0.0547 (7)	
O4	0.5674 (3)	−0.00057 (13)	0.62846 (12)	0.0595 (7)	
S1	0.28433 (9)	0.19677 (4)	0.68662 (3)	0.03081 (19)	

### Atomic displacement parameters (Å^2^)

**Table:**

	*U*^11^	*U*^22^	*U*^33^	*U*^12^	*U*^13^	*U*^23^
C1	0.0406 (16)	0.0483 (18)	0.0233 (14)	0.0236 (14)	−0.0020 (12)	−0.0033 (14)
C2	0.0365 (16)	0.0373 (16)	0.0261 (14)	0.0020 (13)	0.0056 (12)	−0.0015 (13)
C3	0.0296 (15)	0.0309 (14)	0.0270 (14)	−0.0002 (12)	0.0055 (11)	−0.0002 (12)
C4	0.0267 (14)	0.0322 (15)	0.0290 (15)	−0.0027 (11)	0.0033 (12)	−0.0024 (12)
C5	0.0344 (15)	0.0448 (17)	0.0295 (15)	−0.0005 (14)	−0.0042 (12)	0.0001 (13)
C6	0.056 (2)	0.0446 (19)	0.0242 (16)	0.0022 (15)	0.0026 (14)	0.0134 (14)
C7	0.0534 (19)	0.0383 (18)	0.0393 (19)	−0.0083 (15)	0.0079 (15)	0.0100 (15)
C8	0.0513 (19)	0.0471 (19)	0.0349 (18)	−0.0214 (16)	0.0037 (15)	0.0031 (15)
C9	0.0496 (18)	0.0467 (19)	0.0279 (15)	−0.0154 (15)	−0.0060 (14)	0.0084 (14)
C10	0.0265 (14)	0.0402 (17)	0.0275 (15)	0.0008 (12)	0.0057 (11)	0.0011 (12)
C11	0.0452 (17)	0.0461 (19)	0.0293 (16)	0.0124 (14)	−0.0042 (14)	0.0062 (14)
C12	0.059 (2)	0.0433 (19)	0.0368 (18)	0.0120 (16)	−0.0016 (16)	0.0137 (16)
C13	0.0474 (19)	0.0432 (18)	0.0350 (17)	−0.0008 (14)	0.0099 (14)	0.0068 (15)
C14	0.0435 (18)	0.0471 (19)	0.0363 (17)	−0.0077 (14)	−0.0042 (14)	−0.0025 (15)
C15	0.0373 (15)	0.0392 (16)	0.0281 (15)	−0.0009 (13)	−0.0026 (12)	0.0021 (13)
C16	0.0394 (17)	0.050 (2)	0.058 (2)	−0.0181 (15)	0.0056 (17)	0.0087 (18)
Cl1	0.0403 (4)	0.0396 (4)	0.0275 (4)	0.0017 (3)	0.0128 (3)	−0.0108 (3)
N1	0.0507 (17)	0.0366 (15)	0.0347 (15)	−0.0014 (13)	0.0048 (13)	−0.0007 (12)
N2	0.0326 (13)	0.0325 (14)	0.0351 (15)	0.0000 (10)	0.0028 (11)	−0.0005 (11)
O1	0.0323 (10)	0.0607 (15)	0.0387 (12)	−0.0070 (10)	−0.0006 (10)	−0.0059 (11)
O2	0.0565 (14)	0.0451 (13)	0.0281 (11)	−0.0041 (10)	0.0182 (11)	0.0042 (10)
O3	0.0564 (15)	0.0482 (14)	0.0597 (17)	−0.0259 (12)	0.0007 (13)	−0.0081 (12)
O4	0.0644 (16)	0.0474 (15)	0.0667 (18)	0.0107 (12)	0.0152 (14)	0.0303 (14)
S1	0.0299 (3)	0.0364 (4)	0.0261 (4)	−0.0028 (3)	0.0059 (3)	0.0004 (3)

### Geometric parameters (Å, °)

**Table:**

C1—C2	1.555 (4)	C10—C11	1.379 (4)
C1—H1A	0.9600	C10—C15	1.392 (4)
C1—H1B	0.9600	C10—S1	1.752 (3)
C1—H1C	0.9600	C11—C12	1.377 (5)
C2—C3	1.539 (4)	C11—H11	0.9300
C2—N1	1.544 (4)	C12—C13	1.400 (5)
C2—Cl1	1.761 (3)	C12—H12	0.9300
C3—N2	1.481 (4)	C13—C14	1.390 (5)
C3—C4	1.524 (4)	C13—C16	1.493 (5)
C3—H3	0.9800	C14—C15	1.373 (4)
C4—C9	1.382 (4)	C14—H14	0.9300
C4—C5	1.384 (4)	C15—H15	0.9300
C5—C6	1.380 (4)	C16—H16A	0.9600
C5—H5	0.9300	C16—H16B	0.9600
C6—C7	1.400 (5)	C16—H16C	0.9600
C6—H6	0.9300	N1—O3	1.207 (3)
C7—C8	1.367 (5)	N1—O4	1.212 (3)
C7—H7	0.9300	N2—S1	1.639 (3)
C8—C9	1.395 (4)	N2—H2A	0.89 (3)
C8—H8	0.9300	O1—S1	1.423 (2)
C9—H9	0.9300	O2—S1	1.432 (2)
			
C2—C1—H1A	109.5	C11—C10—C15	119.8 (3)
C2—C1—H1B	109.5	C11—C10—S1	121.1 (2)
H1A—C1—H1B	109.5	C15—C10—S1	119.1 (2)
C2—C1—H1C	109.5	C12—C11—C10	120.5 (3)
H1A—C1—H1C	109.5	C12—C11—H11	119.8
H1B—C1—H1C	109.5	C10—C11—H11	119.8
C3—C2—N1	105.9 (2)	C11—C12—C13	120.4 (3)
C3—C2—C1	115.5 (2)	C11—C12—H12	119.8
N1—C2—C1	110.5 (2)	C13—C12—H12	119.8
C3—C2—Cl1	110.3 (2)	C14—C13—C12	118.3 (3)
N1—C2—Cl1	103.78 (19)	C14—C13—C16	121.1 (3)
C1—C2—Cl1	110.1 (2)	C12—C13—C16	120.6 (3)
N2—C3—C4	115.3 (2)	C15—C14—C13	121.3 (3)
N2—C3—C2	105.9 (2)	C15—C14—H14	119.3
C4—C3—C2	113.6 (2)	C13—C14—H14	119.3
N2—C3—H3	107.2	C14—C15—C10	119.7 (3)
C4—C3—H3	107.2	C14—C15—H15	120.2
C2—C3—H3	107.2	C10—C15—H15	120.2
C9—C4—C5	119.1 (3)	C13—C16—H16A	109.5
C9—C4—C3	121.5 (3)	C13—C16—H16B	109.5
C5—C4—C3	119.4 (3)	H16A—C16—H16B	109.5
C6—C5—C4	121.4 (3)	C13—C16—H16C	109.5
C6—C5—H5	119.3	H16A—C16—H16C	109.5
C4—C5—H5	119.3	H16B—C16—H16C	109.5
C5—C6—C7	119.0 (3)	O3—N1—O4	123.8 (3)
C5—C6—H6	120.5	O3—N1—C2	117.7 (3)
C7—C6—H6	120.5	O4—N1—C2	118.6 (3)
C8—C7—C6	120.0 (3)	C3—N2—S1	118.6 (2)
C8—C7—H7	120.0	C3—N2—H2A	109 (2)
C6—C7—H7	120.0	S1—N2—H2A	107 (2)
C7—C8—C9	120.5 (3)	O1—S1—O2	119.59 (14)
C7—C8—H8	119.8	O1—S1—N2	107.37 (14)
C9—C8—H8	119.8	O2—S1—N2	105.25 (14)
C4—C9—C8	120.0 (3)	O1—S1—C10	108.79 (14)
C4—C9—H9	120.0	O2—S1—C10	107.97 (14)
C8—C9—H9	120.0	N2—S1—C10	107.24 (13)
			
N1—C2—C3—N2	59.6 (3)	C12—C13—C14—C15	−1.7 (5)
C1—C2—C3—N2	−63.2 (3)	C16—C13—C14—C15	176.7 (3)
Cl1—C2—C3—N2	171.28 (19)	C13—C14—C15—C10	1.2 (5)
N1—C2—C3—C4	−172.9 (2)	C11—C10—C15—C14	−0.4 (4)
C1—C2—C3—C4	64.4 (3)	S1—C10—C15—C14	178.4 (2)
Cl1—C2—C3—C4	−61.2 (3)	C3—C2—N1—O3	50.9 (3)
N2—C3—C4—C9	36.7 (4)	C1—C2—N1—O3	176.7 (3)
C2—C3—C4—C9	−85.7 (3)	Cl1—C2—N1—O3	−65.3 (3)
N2—C3—C4—C5	−141.2 (3)	C3—C2—N1—O4	−128.4 (3)
C2—C3—C4—C5	96.3 (3)	C1—C2—N1—O4	−2.5 (4)
C9—C4—C5—C6	0.2 (5)	Cl1—C2—N1—O4	115.5 (3)
C3—C4—C5—C6	178.1 (3)	C4—C3—N2—S1	80.2 (3)
C4—C5—C6—C7	−1.6 (5)	C2—C3—N2—S1	−153.3 (2)
C5—C6—C7—C8	2.4 (5)	C3—N2—S1—O1	41.9 (3)
C6—C7—C8—C9	−1.8 (5)	C3—N2—S1—O2	170.3 (2)
C5—C4—C9—C8	0.5 (5)	C3—N2—S1—C10	−74.9 (2)
C3—C4—C9—C8	−177.4 (3)	C11—C10—S1—O1	−18.0 (3)
C7—C8—C9—C4	0.4 (5)	C15—C10—S1—O1	163.2 (2)
C15—C10—C11—C12	0.0 (5)	C11—C10—S1—O2	−149.2 (2)
S1—C10—C11—C12	−178.8 (3)	C15—C10—S1—O2	32.0 (3)
C10—C11—C12—C13	−0.4 (5)	C11—C10—S1—N2	97.8 (3)
C11—C12—C13—C14	1.2 (5)	C15—C10—S1—N2	−80.9 (2)
C11—C12—C13—C16	−177.1 (3)		

### Hydrogen-bond geometry (Å, °)

**Table:**

*D*—H···*A*	*D*—H	H···*A*	*D*···*A*	*D*—H···*A*
C3—H3···O1	0.98	2.46	2.933 (4)	109
C3—H3···O3	0.98	2.42	2.779 (4)	101
C1—H1A···O2^i^	0.96	2.52	3.369 (4)	147
C1—H1B···O1^ii^	0.96	2.58	3.310 (4)	133
N2—H2A···O2^i^	0.89 (2)	2.32 (3)	3.141 (3)	153

Symmetry codes: (i) *x*+1/2, *y*, −*z*+3/2; (ii) *x*+1, *y*, *z*.

## References

[bb1] Allen, F. H., Kennard, O., Watson, D. G., Brammer, L., Orpen, A. G. & Taylor, R. (1987). *J. Chem. Soc. Perkin Trans. 2*, pp. S1–19.

[bb2] Bruker (2000). *SMART* (Version 5.625), *SAINT* (Version 6.01), *SHELXTL* (Version 6.10) and *SADABS* (Version 2.03). Bruker AXS Inc., Madison, Wisconsin, USA.

[bb3] Han, J., Zhi, S., Wang, L., Pan, Y. & Li, G. (2007). *Eur. J. Org. Chem.* pp. 1332–1337.

[bb4] Kemp, J. E. G. (1991). *Comprehensive Organic Synthesis*, Vol. 3, edited by B. M. Trost & I. Fleming, pp. 469–513. Oxford: Pergamon Press.

[bb5] Li, G., Saibabu, K. S. R. S. & Timmons, C. (2007). *Eur. J. Org. Chem.* pp. 2745–2758.

[bb6] Orlek, B. S. & Stemp, G. (1991). *Tetrahedron Lett.* **32**, 4045–4048.

[bb7] Qui, J. & Silverman, R. B. (2000). *J. Med. Chem.* **43**, 706–720. 10.1021/jm9904755 10691696

[bb8] Spek, A. L. (2003). *J. Appl. Cryst.* **36**, 7–13.

